# Dysregulated Cell Signaling in Pulmonary Emphysema

**DOI:** 10.3389/fmed.2021.762878

**Published:** 2022-01-03

**Authors:** Chih-Ru Lin, Karim Bahmed, Beata Kosmider

**Affiliations:** ^1^Department of Microbiology, Immunology, and Inflammation, Temple University, Philadelphia, PA, United States; ^2^Center for Inflammation and Lung Research, Temple University, Philadelphia, PA, United States; ^3^Department of Thoracic Medicine and Surgery, Temple University, Philadelphia, PA, United States

**Keywords:** lung, alveolar epithelium, alveolar type II cells, emphysema, oxidative stress, tissue homeostasis

## Abstract

Pulmonary emphysema is characterized by the destruction of alveolar septa and irreversible airflow limitation. Cigarette smoking is the primary cause of this disease development. It induces oxidative stress and disturbs lung physiology and tissue homeostasis. Alveolar type II (ATII) cells have stem cell potential and can repair the denuded epithelium after injury; however, their dysfunction is evident in emphysema. There is no effective treatment available for this disease. Challenges in this field involve the large complexity of lung pathophysiological processes and gaps in our knowledge on the mechanisms of emphysema progression. It implicates dysregulation of various signaling pathways, including aberrant inflammatory and oxidative responses, defective antioxidant defense system, surfactant dysfunction, altered proteostasis, disrupted circadian rhythms, mitochondrial damage, increased cell senescence, apoptosis, and abnormal proliferation and differentiation. Also, genetic predispositions are involved in this disease development. Here, we comprehensively review studies regarding dysregulated cell signaling, especially in ATII cells, and their contribution to alveolar wall destruction in emphysema. Relevant preclinical and clinical interventions are also described.

## Introduction

**Figure 1:**
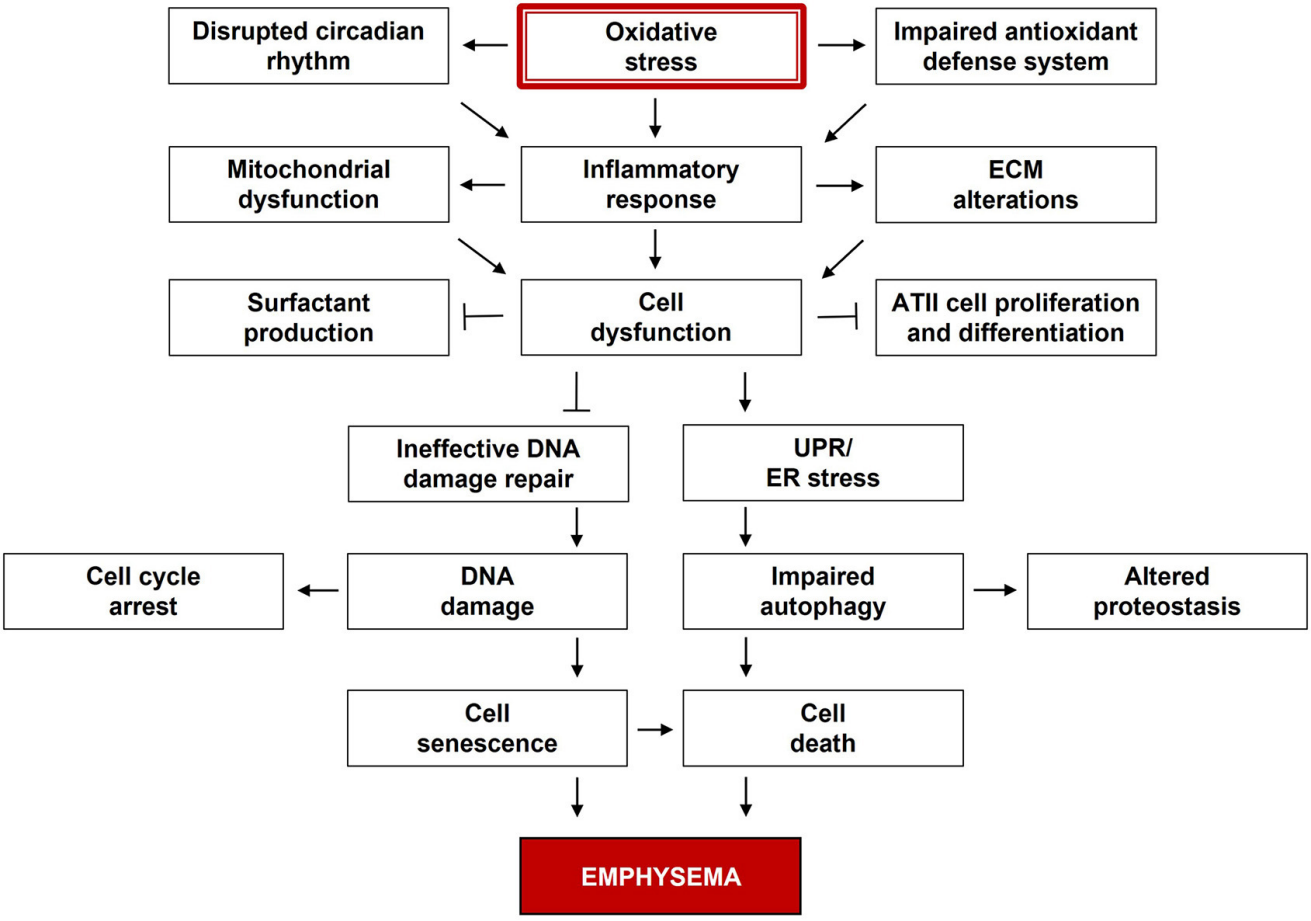
Multiple dysregulated signaling pathways in the pathogenesis of emphysema. Oxidative stress is the major contributor to emphysema. Reactive oxygen species (ROS) and related species disturb cell signaling and impair cell functions, while the antioxidant defense system is overwhelmed. These components are dynamically and progressively interactive over time, and their alterations can lead to emphysema development.

Over 300 million people suffer from chronic obstructive pulmonary disease (COPD) worldwide (1). It is the third leading cause of death, resulting in ~3 million deaths every year, according to World Health Organization (WHO) (2). COPD includes lung parenchymal destruction (emphysema) and airway disease (chronic bronchitis). The extent of lung tissue damage in emphysema is measured by chest computed tomography (CT) density. Emphysema is a progressive and irreversible disease with limited therapeutic strategies. Lung volume reduction surgery and lung transplantation represent promising options for end-stage disease (3). At the cellular level, emphysema is characterized by alveolar epithelial cell death and impaired re-epithelialization (Figure 1), which causes alveolar wall destruction and decreased surface area in the lung parenchyma for gas exchange (4). Pulmonary vasculature is linked to alveolar architectures and function, whereas endothelial dysfunction and vascular abnormalities were observed in emphysema (5, 6). Furthermore, extracellular matrix (ECM), including elastin, collagen, and proteoglycans, tethers the parenchymal compartments to the airway and affects the tissue mechanics and airway smooth muscle contraction. The process of mechanotransduction provides mechanical cues of the microenvironment to control many cellular events such as proliferation and differentiation and maintain tissue integrity (7). Changes in ECM composition in emphysema may impact airway smooth muscle cells function, including hypertrophy and hyperplasia, resulting in airway remodeling and obstruction. Also, the loss of elastic recoil leads to airspace enlargements and irreversibly weakens respiratory airflow (8). Increased lung volume, gas trapping, and reduced alveolar units are observed in emphysema patients compared to controls (4).

Environmental irritants, especially cigarette smoke, are the main risk factors of emphysema development. Cigarette smoke contains thousands of chemicals and oxidants that can induce oxidative stress and modify biomolecules, including proteins, lipids, nucleic acids, or carbohydrates, thus affecting lung physiology and tissue homeostasis (9). Surfactant is essential for alveoli and smoking has adverse effects on its function and composition through oxidation and altered proteostasis (10). For instance, Takamiya et al. showed that cigarette smoke extract and its component acrolein modified the surfactant protein (SP)-A in the alveolar epithelium (11). The ability of SP-A to inhibit bacterial growth and macrophage phagocytosis *in vitro* was attenuated due to its conformational changes. The antioxidant defense systems can protect from damage, whereas they are overwhelmed by persisting oxidative stress (9). Cigarette smoke-induced reactive oxygen species (ROS) disturb cell signaling and impair cell functions, including inflammatory responses and protease-antiprotease balance, resulting in ECM destruction and alveolar epithelial cell injury and death (12). Furthermore, emerging senescent cells and vascular dysfunction in the lungs fail the microenvironment for alveolar re-epithelialization.

Besides environmental factors, genetic susceptibility has a significant impact on the morbidity of COPD, such as alpha-1 antitrypsin (AAT) deficiency (13). Studies have identified multiple genetic loci related to this disease susceptibility, e.g., *HHIP, FAM13A, IREB2, MMP12, MMP1, RAGE, SFTPD, FBLN5* in humans and mice (14). These genes are associated with surfactant function, tissue growth, remodeling, and homeostasis. The large complexity of lung pathophysiological processes implicated in emphysema contributes to its heterogeneity. Genetic susceptibility, age, sex, and race-dependent differences may also contribute to this disease development.

## Dysregulation of Cell Signaling in Emphysema Development

### Oxidative Stress

Cigarette smoke-induced oxidative stress and redox imbalance are major contributors to emphysema (15). ROS and related species act as second messengers in cell signal transduction and participate in cellular physiological responses, including the inflammatory immune system and mitochondrial respiration. Oxidative stress refers to high ROS levels that overwhelm the antioxidant defense system. It can alter the activities and functions of redox-sensitive molecules and metabolic enzymes such as p53, NRF2, NF-κB, JNK, MAPK, protein tyrosine phosphatases, glutathione, thioredoxin, peroxiredoxins, and histone deacetylase (15, 16). High oxidative stress and the defective antioxidant defense system were detected in the lungs and alveolar type II (ATII) cells in emphysema patients (15, 17). Especially reduced NRF2 (18) and FOXO3 (19) levels were observed, which are important transcription factors regulating multiple antioxidant genes such as *HO-1, NQO1*, and *GPX1*. Also, NF-E2 was downregulated in ATII cells in this disease (20). Several NRF2 activators and different classes of antioxidants have been developed and tested in clinical trials, however, without promising results (15). Increasing evidence shows that the small redox protein thioredoxin relieved animal emphysema and pulmonary inflammation through multiple mechanisms (21). Its therapeutic potential was recently proposed.

We have demonstrated that DJ-1 protein modulates the NRF2-mediated antioxidant defense system in human primary ATII cells after exposure to cigarette smoke (22). Altered DJ-1 function, its overoxidation to sulfonate form (-${\text{SO}}_{3}^{-}$) at the cysteine-106 and ubiquitination, were reported in the pathophysiological response to oxidative damage and emphysema patients (17). Of note, DJ-1 oxidation to sulfinate form (-${\text{SO}}_{2}^{-}$) was detected in smokers, which reflected its cytoprotective activity. Oxidative stress-induced DJ-1 overoxidation and cleavage in mitochondria were observed in A549 cells treated with hydrogen peroxide. DJ-1 ablation resulted in mitochondrial dysfunction. We also showed impaired S100A8 function in ATII cells in emphysema (23). S100A8 belongs to the S100 protein family and responds to oxidative stress. ATII cell death in this disease correlated with decreased S100A8 sulfination (24). Furthermore, the cytoprotective function of S100A8 compensated for the absence of DJ-1 *in vitro* and *in vivo*. Targeting redox regulation appears to be an ideal approach to this disease yet challenging, possibly due to delicate redox balance, disparities between animal models and human diseases, species differences, compound bioavailability, and other variables.

### Pro-inflammatory Response

Environmental irritants can promote the recruitment of inflammatory cells into the lungs. The pro-inflammatory and immune responses are evident in emphysema pathophysiology. Glucocorticoids and bronchodilators are commonly used to treat COPD, although both have many side effects, including immunosuppression (21). The PDE4 inhibitor roflumilast is an approved drug that selectively inhibits PDE4 and increases cAMP levels, leading to an anti-inflammatory response. Numerous PDE inhibitors are in clinical testing. Dual PDE3/4 inhibitors such as RPL554 have gained interest in enhanced efficacy as PDE3 is expressed in vascular smooth muscle cells, and its inhibitor induces bronchodilation (25). Similarly, drug combinations may have a synergistic effect over mono-components. For example, combination of PDE4 and PI3Kδ inhibitors significantly increased protection against cigarette smoke extract-induced apoptosis of lung epithelial cells and reduced inflammatory responses of neutrophils and macrophages *in vitro*.

Under normal conditions, the immune cells balance lung defense, tolerance, or tissue repair. Particularly, alveolar macrophages (AM) interact with pulmonary surfactants in the innate immune responses. AM are potent phagocytes, regulate adaptive immunity and recruit neutrophils and monocytes into the lungs. They secrete inflammatory mediators and proteases such as ECM degrading enzymes and cathepsins upon activation, resulting in elastolysis, alveolar tissue damage, and remodeling of alveoli (8, 12). AM show plasticity based on the microenvironment. Lechner et al. indicated that M2-like macrophages and bone marrow-derived monocytes constitute a regenerative ATII cell niche component, modulating their proliferation and differentiation (26). Using knockout mice and adoptive transfer studies, they found that macrophages accumulated in the lung *via* the CCL2-CCR2 chemokine recruitment axis during lung regeneration after pneumonectomy. F4/80+ M2-like macrophage and ATII cell co-culture supported the formation of pneumospheres.

Nevertheless, high inflammation in smokers correlated with emphysema development (8, 27). Inflammatory responses downregulated *SFTPB, SFTPD, SCNN1A*, and *SCNN1B* gene expression, related to surfactant production and alveolar fluid clearance in ATII cells (28). Decreased SP-D expression was detected in the lungs of emphysema patients (29), indicating the defects in ATII cell function and innate immunity. Especially the number of AM is negatively related to alveolar parenchymal tissue density (27), and impaired phagocytosis is positively associated with the severity of emphysema (30). Increased microRNA (miR)-155 expression was recently identified in the smokers' lungs and in AM of mice exposed to cigarette smoke (31), contributing to inflammation and this disease development. ATII cells isolated from subjects with this disease showed high oxidative stress (17, 32) and ECM degradation with the elevation of matrix metalloproteinase (MMP) 9, CD147, and cathepsin B (20). Other proteases elevated in patients with emphysema include neutrophil elastase, dipeptidyl peptidase-4, proteinase-3, MMPs 1, 8, 12, and 13, cathepsins C, D, E, G, K, and S, and caspases 1, 3, 7, 8, 9, and 11 (33). Several studies have shown the efficacy of antiprotease therapy in emphysema; however, without promising results in clinical trials (21). On the other hand, ADAM8 belongs to a disintegrin and metalloproteinase family. It was reported as a protective proteinase negatively related to emphysema development (34). ADAM8 levels were decreased in macrophages and alveolar epithelial cells in patients with this disease and mice exposed to cigarette smoke. Moreover, ADAM8 knockout mice displayed higher inflammatory and oxidative stress levels, mucus cell metaplasia, alveolar septal cell death, and reduced ATII cell proliferation and repair, contributing to the lung destruction caused by cigarette smoke exposure. Accordingly, pro-inflammatory mediators, oxidative stress, protease-antiprotease imbalance, and impaired pathogen phagocytosis contribute to the destruction of lung tissue and this disease progression (12). Many anti-inflammatory drugs have been developed but have limited efficacy (21). Further studies of immune responses and homeostasis can provide a clue for therapeutic interventions and lung regeneration.

Increasing evidence indicates that the nuclear hormone receptor PPARγ, a regulator of lipid metabolism, adipogenesis, and inflammation, represents a potential therapeutic target for emphysema. It was downregulated in antigen-presenting cells (APCs) isolated from the lungs of patients with this disease and mice exposed to cigarette smoke, thus directing Th1 and Th17 cell differentiation (35, 36). Also, overexpression of dominant-negative PPARγ in murine ATII cells induced emphysema with increased inflammatory cytokines, MMPs, and accumulation of myeloid-derived suppressor cells (MDSCs) in the lungs and circulation system (37). As expected, treatment with a PPARγ agonist ciglitazone inhibited pathogenic lung APCs *in vitro* in humans and mice. Further, it attenuated cigarette smoke-induced emphysema in mice (35, 36). Thiazolidinediones (TZDs), including ciglitazone, pioglitazone, rosiglitazone, and troglitazone, are valuable drugs for type 2 diabetes and have shown anti-inflammatory effects through selective stimulation of PPARγ. Their application to emphysema needs to be further evaluated.

### Chronic Inflammation

Chronic inflammation can increase susceptibility to lung infection. Cigarette smoke-induced emphysema in animals was challenged with bacterial or viral infections to study the mechanism of this disease exacerbation (38). H3N1 influenza A virus infection caused higher virus titers in the lung, inflammation around bronchi and in the parenchyma, and mucus exudates in the airways in cigarette smoke-exposed mice compared to the influenza virus alone (39). Also, *Pseudomonas aeruginosa* infection resulted in impaired autophagy with an increased number of aggresomes, cell senescence, and alveolar space enlargement in mice exposed to cigarette smoke compared to controls (40). Cysteamine, an antioxidant drug with autophagy-inducing properties, alleviated these changes. *Nippostrongylus brasiliensis*, a nematode in rodents, was used to develop a mouse model of progressive emphysema (38). This infection induced a long-lasting Th2 immune response and alternatively activated M2 macrophages accompanied by substantially upregulated MMP12 expression, resulting in alveolar wall destruction and airspace enlargement. Increasing evidence shows the formation of autoimmune components, including autoantibodies and key mediators (such as BAFF and IL-17A), increased B cell counts, and B cell-rich lymphoid follicles in emphysema patients, which were associated with this disease severity (41, 42). Zhou et al. exposed mice to 150–180 mg/m^3^ cigarette smoke for 2 weeks followed by 2 weeks of rest before the elastin challenge to study the autoimmunity in emphysema development (43). MMP12-generated elastin fragments are self-antigens that induce monocyte chemotactic activity and contribute to this disease pathology. Exposure to cigarette smoke sensitized mice to elastin and elicited IL17A-predominant autoimmune processes leading to neutrophilic airway inflammation, mucus hyperproduction, airspace enlargement, and lung function decline. Of note, elastin-specific T cell response was also observed in COPD patients. The correlation of B cell adaptive immune responses with late-stage emphysema introduces opportunities for new therapeutic interventions. However, the crucial role of B cells in the defense against pathogenic infections and immune-regulatory activities limits anti-B cell therapies. Further characterization of B cell subsets and their contribution to disease progression are needed.

### Altered Cellular Homeostasis

Cigarette smoke-induced oxidative stress increases protein misfolding (44), thus altering lung homeostasis. It induces endoplasmic reticulum (ER) stress and unfolded protein response (UPR), leading to inflammation and cell apoptosis. ER stress was observed in emphysema patients and animals (45). Activation of UPR with the upregulation of GRP78 was detected in human small airway epithelial cells and ATII cells obtained from smokers (46). The aggresome formation, involved in the cellular response to misfolded proteins and their clearance, was also observed in the lungs of smokers, emphysema patients, mice exposed to cigarette smoke, and aged mice (40). Specifically, the levels of aggresome correlated with smoking history and emphysema severity in humans. Cigarette smoke increased aggresome formation by accumulating ubiquitinated proteins and autophagy proteins p62 and valosin-containing protein (VCP) in murine lungs, indicating aberrant autophagy (40). Intraperitoneal administration with the autophagy-inducing drug cysteamine reduced cigarette smoke-induced aggresome formation in the lungs, inflammatory responses in bronchoalveolar lavage fluid (BALF), and emphysematous changes in mice. Similarly, inhibition of ER stress by treatment with 4-phenylbutyric acid reduced cigarette smoke-induced inflammatory response, alveolar cell apoptosis, and airspace enlargement in mice (47). Therefore, the ER stress-autophagy pathway represents a potential therapeutic intervention.

Several studies have pointed out the deleterious impact of ceramides or lactosylceramides accumulation in the human lung in emphysema progression (48), likely due to impaired autophagy resulting in sphingolipids imbalance. They were accumulated in different cell types, including alveolar epithelial and endothelial cells and macrophages in patients with this disease (49). Ceramides are components of sphingolipids and serve as second messenger lipids. Their upregulation leads to inflammatory responses, which can cause alveolar epithelial cell apoptosis and emphysema development in animal models (49). Cigarette smoke promoted the activation of membrane-localized acid sphingomyelinase, which catalyzes the hydrolysis of sphingomyelin to ceramide. This thereupon increased membrane and intracellular ceramide accumulation in aggresomes (48). Cysteamine reduced cigarette smoke-induced ceramide accumulation in Beas2b cells (48). It improved efferocytosis, cell viability, and decreased cell senescence. Also, intraperitoneal injection of autophagy-inducer gemfibrozil significantly reduced ceramide accumulation in murine lungs after cigarette smoke exposure.

### Circadian Rhythm Disruption

The human respiratory system functions with a daily circadian rhythm, affecting airway resistance, ventilatory controls, and immune function, yet it changes with age and smoking (50, 51). Smokers have marked diurnal changes in pulmonary function tests compared to non-smokers (51). The lungs' molecular clock is also altered in response to environmental factors, including allergens, pollutions, pathogens, infections, oxidative stress, hypoxia/hyperoxia, jet lag, and shift work (52). Importantly, circadian gene expression positively connects to cell cycle and immune regulation in mice and humans (53). Increasing evidence shows a link between circadian rhythm disruption and susceptibility to lung infection (54) and inflammatory diseases (55), including asthma and COPD with severe symptoms in the early morning, which correlated with disease exacerbations.

Pekovic-Vaughan et al. have demonstrated the circadian regulation of the NRF2 pathway in the murine lung, mainly in the bronchial and alveolar epithelium (56). The loss of NRF2-dependent antioxidant defense system and reduced-glutathione levels increased oxidative damage in the lungs of Clock^Δ19^ mutant mice compared to wild-type mice. SIRT-1 functions as NAD^+^-dependent protein deacetylases and regulates many pathophysiological processes. It controls the circadian clock through binding with CLOCK-BMAL1 complexes and promoting the deacetylation and degradation of PER2 proteins (57). In the lungs of mice exposed to cigarette smoke, altered rhythms of SIRT-1 protein and core clock genes including *Bmal1, Clock, Per1, Per2, Cry1, Cry2, Nr1d1, Nr1d2*, and *Rora* were observed in a circadian manner, increasing the susceptibility to inflammation and emphysema development (58). Similarly, BMAL1 protein was down-regulated in the lungs of smokers and individuals with this disease (58). BMAL1 deficiency accelerated senescence and caused age-associated shrinkage of major organs, including lungs, in 40-week-old mice, which correlated with increased ROS levels and inflammation (59). Melatonin, a natural hormone that regulates sleep-wake cycles, has shown beneficial effects on lung diseases through anti-oxidation and anti-inflammaton (60). It increased SIRT-1 expression *in vitro* and *in vivo* (61, 62). Intraperitoneal injection with melatonin decreased inflammatory response in BALF, and the lungs of mice challenged with cigarette smoke and lipopolysaccharide (61). It also reduced ER stress and bronchial and alveolar epithelial cell apoptosis, and protected alveolar architecture in a rat model of emphysema (62). The daily rhythm of SP-A, SP-B, and SP-C gene expression in the rat lungs was reported (63). This indicates the critical role of circadian rhythm in ATII cell function; however, its alterations in lung pathophysiology are still poorly understood.

### Cellular Senescence

Cigarette smoke can cause DNA damage, while the impairment of double-strand DNA break repair in ATII cells was shown in emphysema patients (32). Oxidative stress and persistent DNA damage are associated with stress-induced premature senescence (SIPS) (64). SIPS induces irreversible cell cycle arrest, chromatin changes, and resistance to apoptosis. It also drives a senescence-associated secretory phenotype (SASP) in cells, including secretion of inflammatory cytokines IL-6 and IL-8 and remodeling factors, thereby affecting tissue microenvironment and promoting senescence in an autocrine and paracrine manner (65). DNA damage response (DDR) is required for SASP, including cell cycle regulatory protein NBS1 and checkpoint kinases ATM and CHK2 (66). Transient exposure to SASP promoted stem cell function and tissue regeneration, whereas prolonged exposure had an opposite effect in mice (67). Mice with GFP-expressing ATII cells (CBA/Ca × C57BL6J) exposed to cigarette smoke for over 3 months displayed airspace enlargement and alveolar epithelial cell apoptosis (68). The surviving ATII cells showed higher resistance to apoptosis, which was likely related to circadian rhythm. This suggests the contribution of ATII cell senescence and the circadian clock's regulation to emphysema development. Reduced anti-aging protein SIRT-1 levels were found in the lungs of smokers and patients with emphysema, including AM, airway, and alveolar epithelium (69, 70). Particularly, oxidative stress-induced miR-34a expression was detected in lung tissue in these patients leading to suppression of SIRT-1 and SIRT-6 expression *via* the PI3K pathway (70). Furthermore, increased lipofuscin levels, a marker of senescence, and cell cycle inhibitors p16 and p21 were mainly detected in ATII cells and endothelial cells in emphysema patients compared to controls (71). Telomere signal intensity in these cells was lower in smokers and emphysema than in controls. A negative correlation between p16 and proliferation cell nuclear antigen (PCNA) was also reported. Similarly, other cells displayed characteristics of cellular senescence, including airway epithelial cells, fibroblasts, and immune cells (72). Their accumulation indicates the loss of tissue homeostasis and an altered environment for alveolar re-epithelialization in emphysema. Senotherapies are effective in animal models, including SIRT-1 activators and inhibitors of mTOR, JAK, FOXO4, and anti-apoptotic proteins (72). Drugs targeting anti-apoptotic proteins in senescent cells such as dasatinib and quercetin were well-tolerated in patients with age-related diseases. Senotherapy seems promising since the mechanism of cellular senescence is likely shared between different diseases.

Furthermore, mitochondria generate ATP for cell metabolism, and their dysfunction has been linked to age-related lung diseases. Several mitochondria-targeted antioxidants, including mitoQ, mito-TEMPO, pyrroloquinoline quinone, and SkQl are in clinical trials for other age-related diseases (72). Increased mitochondrial (mt)DNA copy number has been detected in the blood of patients with COPD along with a positive correlation to the number of pack-years smoking (73). It was also found in urine and was associated with respiratory symptoms and emphysema severity (74). Importantly, elevated mitochondrial ROS levels, low mitochondrial amount, accumulation of mtDNA damage, and mitochondrial dysfunction were detected in human ATII cells in this disease progression (75). It has been shown that electronic (e)-cigarette aerosols containing nicotine caused airway hyperreactivity, alveolar destruction, reduced lung function, and emphysematous changes in mice (76). Nicotine altered calcium levels and mitochondrial membrane potential in human primary ATII cells with DJ-1 knockdown (77). Dysregulation of mitochondrial oxidative phosphorylation (OXPHOS) complexes was observed in the lungs of DJ-1 knockout mice exposed to aerosolized nicotine, which disrupted the nuclear/mitochondrial stoichiometry resulting in mitochondrial dysfunction. This was associated with increased AM number and ATII cell apoptosis. Both ER stress and mitochondrial dysfunction were detected in emphysema, however, with unknown integration. It was recently demonstrated that ER stress led to mitochondrial UPR in an ATF4-dependent manner in MLE-12 cells (78). Mitochondrial UPR has contributed to different disease pathogenesis (79), although its activation is also known to lifespan extension in both *Caenorhabditis elegans* and mice (80). Improving our understanding of the mitochondrial role in cellular senescence can greatly benefit treating emphysema.

### Apoptosis

The correlation of alveolar cell apoptosis and decreased lung surface area have been shown in emphysema (81). Intratracheal instillation of active caspase-3 protein led to alveolar epithelial cell apoptosis and this disease development in mice (82). Increased active caspase 3 levels were detected in ATII cells in smokers and individuals with emphysema (24). Histone deacetylase inhibition altered chromatin remodeling leading to ATII cell apoptosis and alveolar structure destruction in trichostatin A-treated rats through increased miR34a, p53, cleaved caspase 3, and microtubule-associated protein-1 light chain 3 (LC3) levels (83). Decreased hypoxia-inducible factor-1α (HIF-1α), vascular endothelial growth factor (VEGF), lysyl oxidase, and collagen expression in this animal model pointed out the involvement of angiogenic factors in the alveolar structure. Subcutaneous injection of VEGF receptor (VEGFR) blocker led to emphysema without the infiltration of inflammatory cells in rats (84). Treatments targeting VEGF signaling have beneficial effects against emphysema development in animal models (85–87). Alveolar and endothelial cells can sense microenvironment changes; therefore, they serve as niches for regulating lung repair and integrity. Angiogenesis impairment by subcutaneous injection of sodium glutamate enhanced lung inflammation and emphysema in mice induced by intratracheal instillation of cigarette smoke extract, which was related to insufficient migration of pericytes and smooth muscle cells in lung tissue (88). Surprisingly, intravenous administration of healthy lung endothelial cells ameliorated emphysematous phenotype and lung function in an elastase-induced mouse model (89). It was recently reported that endothelial cells released angiocrine sphingosine-1-phosphate (S1P) after *Pseudomonas aeruginosa* infection in mice which promoted alveolar re-epithelialization *via* S1PR2-YAP signaling (90). Supplementation of S1P prevented alveolar cell apoptosis in VEGFR blockade-induced emphysema in mice (91). This suggests that sphingolipid balance is important for the maintenance of alveolar septal integrity.

### Smooth Muscle Cell Proliferation

In addition to endothelial dysfunction, pulmonary vascular remodeling characterized by increased smooth muscle cell proliferation and narrowing of the vascular lumen has been shown in smokers and to precede airspace enlargement in animal models of emphysema (92). It can subsequently lead to vascular wall thickening, pulmonary hypertension, and right heart failure. Bronchodilators that target the smooth muscles were beneficial in reducing COPD exacerbation risk vs. placebo in large-scale randomized trials (93). Significant improvements in forced expiratory volume (FVC) were reported after bronchodilator administration in subjects with emphysema (95, 96). Treatment with complementary bronchodilators or a combination of inhaled corticosteroids with bronchodilators was more effective than monotherapy (94). Pharmacological lung volume reduction through bronchodilator therapy is an important goal in these patients.

Smooth muscle cells are the mechanical sculptor of the epithelium (97). Nitric oxide (NO) production by nitric oxide synthase (NOS) regulates the degree of contraction of vascular smooth muscle cells and their proliferation. ATII cells express constitutive NOS as well as inducible NOS (iNOS) during inflammatory states, suggesting their interaction with vascular smooth muscle cells. The absence of SP-D in mice displayed iNOS-related chronic inflammation, alterations of surfactant homeostasis, and emphysematous changes. This elucidates the contribution of immune responses to NO/iNOS regulation (98). iNOS inactivation by genetic deficiency and pharmacological inhibition prevented cigarette smoke-induced pulmonary hypertension and emphysema, including structural and functional alterations of the lung vasculature and alveoli in mice (99). In clinical studies, elevated alveolar NO has been associated with COPD severity (100). These patients have higher numbers of iNOS^+^ ATII cells related to increased protein nitration and decreased lung function (101). Especially, the lung tissue from patients with emphysema has a higher ratio of the number of alveoli/vessels and an increased degree of vessel muscularization. Nitrated proteins in vasculature and alveoli were increased in these patients (99). NO can be toxic through combination with superoxide to generate peroxynitrites leading to nitration of biomolecules, altering their structure and function. Further, peroxynitrites induced alveolar epithelial and endothelial cell apoptosis (99). However, chronic exposure to the NOS inhibitor N^ω^-nitro-L-arginine methyl ester resulted in vascular senescence, hypertension, and emphysema development in mice (102). This emphasizes the importance of NO/NOS balance in vascular health and alveolar structure.

Chronic hypoxia in emphysema induces pulmonary artery smooth muscle cells proliferation and JAK2/STAT3 activation. JAK2 deficiency attenuated pulmonary vascular remodeling and smooth muscle hyperplasia in mice (103). Furthermore, HIF-1α promotes vascular smooth muscle cell proliferation under hypoxic conditions. Its increased expression was detected by immunohistochemistry in the lungs of emphysema patients, indicating vascular remodeling in this disease. Especially, HIF-1α was positively associated with disease severity (104). With oxygen treatment, the HIF-1α and erythropoietin decreased in COPD (105). It has been demonstrated that HIF-1α signaling in ATII cells promoted their proliferation after acute lung injury in mice induced by lipopolysaccharide or hydrochloric acid (106). ATII cell-specific HIF-1α deletion caused higher mortality in these mice. Also, HIF-1α is activated in the ATII to alveolar type I (ATI) cell transitional state (107). Together, these data show the intimate relationship between alveolar epithelium and smooth muscle cells for alveolar architecture and function.

### Dysregulated Alveolar Re-epithelialization

ATII cells are progenitors in alveoli. Various ATII niches, mediators, and signaling support their functions for alveolar epithelial cell homeostasis and re-epithelialization. The interactions between signaling molecules arising from and acting on the alveolar epithelium, vasculature, mesenchyme, ECM, and immune cells institute ATII cell niches. Dynamic organization of WNT/β-catenin, TGF-β, YAP/TAZ, NOTCH, and TP53 signaling pathways participate in ATII cell growth and differentiation to ATI cells (108). The importance of WNT/β-catenin, YAP/TAZ, and TGF-β signaling in emphysema development has been demonstrated in animal models. Specifically, *TGF-*β*2* was identified as a significant emphysema-associated genetic variant by human genome-wide association studies (GWAS) (109). TGF-β regulates multiple context-dependent cellular processes associated with tissue remodeling and is crucial for epithelial-mesenchymal interactions. It includes the regulation of cell polarity, ECM turnover, ATII to ATI cell transdifferentiation, and differentiation of lung fibroblasts to myofibroblasts positive for α-smooth muscle actin (α-SMA). Leucine-rich α-2-glycoprotein-1 (LRG1), known to regulate TGF-β signaling, was increased in endothelial cells in patients with emphysema and correlated to its severity (89). Elevated YAP protein levels were detected in the lungs of patients with this disease compared to healthy donors (110). In contrast, reduced WNT/β-catenin pathway in ATII cells was observed in emphysema patients (111, 112) while the non-canonical WNT pathway was upregulated (113, 114). These data suggest signaling imbalance and ATII cell dysfunction in the emphysematous lung. It is known that WNT/β-catenin and anti-inflammatory PPARγ signaling pathways are integrated, and they work in opposition, highlighting the relationship between immune response and ATII cell fate. Feller et al. demonstrated that a non-canonical WNT ligand WNT5a and pro-inflammatory cytokines can be transported through extracellular vesicles, leading to systemic inflammation in COPD patients (115). This further points out the importance of the interactive signaling and microenvironment in the pathogenesis of this disease.

HHIP is a component of hedgehog signaling, which is important in many developmental processes (116). It is a genetic locus associated with emphysema susceptibility in humans and mice. Sonic hedgehog signaling is required for myofibroblast differentiation and mesenchymal proliferation during alveologenesis, while it maintains quiescence in the adult murine lung (117). A broad population of hedgehog-receptive mesenchymal cells surrounding airways and alveoli was identified in the murine lung (118). The transgenic activation of the hedgehog in the mesenchyme disrupted the alveolar niche. This impaired ATII cell proliferation, increased airspace, and emphysematous changes. Single-cell transcriptome analysis of the human lung shows that mesenchymal subsets are conserved across species. The intermediate mesenchymal subset was involved in cholesterol metabolism, suggesting its role in surfactant biosynthesis of the alveolar epithelium. In addition, Kato et al. demonstrated that the paracrine signaling capabilities of pericytes, specialized mesenchymal cells surrounding the capillary, are crucial for alveologenesis. Loss of pericyte-specific YAP/TAZ reduced endothelial and ATII cell proliferation through paracrine regulation, resulting in defective alveolarization and a severe emphysema-like morphology in mice (119). It changed the growth factor expression profiles of these cells in the lungs, including reduced *Angpt1, Tgfb2, Wnt11, Bmp4*, and *Hgf* (119). Treatments targeting ATII cell proliferation and differentiation to ATI cells have not been developed owing to plenty of unknowns regarding the mechanism of alveolar re-epithelialization.

### Signaling Interplay

Oxidative stress, ER stress response, and inflammation are key contributors to emphysema development. Their interactive relationships have been reported (120). ROS provide an oxidizing environment and affect molecular chaperones and enzymes in the ER. In response to ER stress, the UPR helps cells adapt to and survive the stress condition by transcriptional and translational reprogramming. In contrast, programmed cell death signaling is activated when protein homeostasis cannot be achieved. UPR signaling is initiated by ER membrane-bound transducers: IRE1, ATF6, and PERK. Particularly, IRE1α regulates many cell functions, including metabolism, immunity, inflammatory cytokine production, cell differentiation, and apoptosis, through the RIDD mechanism, which induces the degradation of certain mRNAs or miRNAs. Of note, the IRE1α-mediated XBP1 pathway is essential for optimal expression of inflammatory cytokines and proangiogenic factors in human aortic endothelial cells (121, 122). Under chronic or severe ER stress, PERK-mediated phosphorylation of eIF2α induces expression of ATF4 and a proapoptotic factor CHOP/GADD153, resulting in ER stress-induced apoptosis. ATF4 can also induce growth arrest and DNA damage-inducible protein GADD34. Furthermore, ER and mitochondria are physically and functionally connected through mitochondria-associated ER membranes, which contain major calcium channels IP3R and VDAC. ROS can target ER calcium channels, leading to ER calcium release. It subsequently stimulates mitochondrial metabolism and ROS production. The delicate balance between different cell signaling can be the key to therapeutic benefit.

Given that ROS is involved in various physiological processes, the interplay between signaling potentially causes a cascade of cell dysfunction and imbalance under pathological stimuli, leading to tissue damage. Sirtuins (SIRT1-7) maintain cellular redox balance and are an important defense mechanism against oxidative stress according to the different subcellular localization of each sirtuin. Specifically, the ability of SIRT1 to intersect with different signaling, including the circadian clock, inflammation, cell cycle, and senescence, makes it a lucrative target for therapeutics. SIRT1 activators include baicalin, exendin-4, ginkgolide-B, pitavastatin, quercetin, SRT2104, and vitamin D. These medications are in clinical trials for other diseases. Of note, exendin-4 is a licensed medicine for patients with type 2 diabetes. Exendin-4 treatment improved lung function, mortality, and clinical appearance in mice with lipopolysaccharides-induced emphysema, possibly through bronchodilatory effects (123). The mechanistic links between diabetes and COPD imply the applicability of this medicine (124).

## Conclusion

Despite numerous investigations, therapeutic strategies for pulmonary emphysema remain limited mainly due to the complexity and heterogeneity of disease manifestation. Multiple dysregulated signaling pathways affect physiological processes in emphysema development (Figure 1). Its pathological features include high inflammatory and immune response, oxidative stress, defective antioxidant defense system, altered protein homeostasis, cellular senescence, and apoptosis. Circadian rhythm disruption leads to abnormal cell cycle and immune dysregulation, which points out a poor lung repair in this disease. These components are dynamically and progressively interactive over time. Individual differences and genetic variations in surfactant function, tissue growth, remodeling, and homeostasis can also contribute to this disease development. Targeting the cellular processes to decrease alveolar injury and increase alveolar re-epithelialization may restore lung function. Various factors and signaling are interdependent in complex pathophysiology. Therefore, desired outcomes could be achieved by targeting multiple pathways. More systematic and comprehensive studies regarding interactions between different cell types, organelles, and signaling pathways are warranted to uncover new therapeutic strategies.

## Author Contributions

C-RL performed the literature search, prepared the figures, and wrote the manuscript. KB and BK provided significant works of literature, interpretations, and revision. All authors contributed to the article and approved the submitted version.

## Funding

This work was supported by the American Heart Association Postdoctoral Fellowship 20POST35210318 (C-RL), R21 ES030808, Department of Defense W81XWH2110400, and the Catalyst Award from American Lung Association (KB), R01 ES032081, R01 HL150587, and the Department of Defense W81XWH2110414 (BK).

## Conflict of Interest

The authors declare that the research was conducted in the absence of any commercial or financial relationships that could be construed as a potential conflict of interest.

## Publisher's Note

All claims expressed in this article are solely those of the authors and do not necessarily represent those of their affiliated organizations, or those of the publisher, the editors and the reviewers. Any product that may be evaluated in this article, or claim that may be made by its manufacturer, is not guaranteed or endorsed by the publisher.
